# Developing odontoma arising from calcifying odontogenic cyst: A case report

**DOI:** 10.1002/ccr3.5011

**Published:** 2021-11-22

**Authors:** Monir Moradzadeh Khiavi, Nazanin Mahdavi, Asoma Awudu

**Affiliations:** ^1^ Department of Oral and Maxillofacial Pathology School of Dentistry Tehran University of Medical Sciences Tehran Iran; ^2^ School of Dentistry International Campus Tehran University of Medical Sciences Tehran Iran

**Keywords:** ameloblastic fibro‐odontoma, calcifying odontogenic cyst, developing odontoma, histopathology, mixed odontogenic tumor, panoramic radiography

## Abstract

Developing odontoma is a rare mixed odontogenic tumor that can arise with other odontogenic lesions. The association of COC with ameloblastic fibro‐odontoma is extremely rare. We report an extremely rare case of developing odontoma arising from a calcifying odontogenic cyst in a 17‐year‐old girl.

## Graphical abstract

The importance of accurate diagnosis that influence the treatment plan. In this case with accurate diagnosis, conservative treatment was substituted with marginal resection.

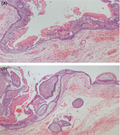

## 1 INTRODUCTION

Developing odontoma replaced ameloblastic fibro‐odontoma (AFO) as a new entity in the latest (4th) edition, 2017, of the World Health Organization (WHO) Head and Neck Tumors Classification.^1^ The World Health Organization has classified developing odontoma as a mixed odontogenic tumor.^2^ It is a rare mixed odontogenic tumor characterized by the proliferation of odontogenic epithelium as cords and small islands in a background of primitive ectomesenchymal cells similar to dental papilla and in association with calcified tooth structures including enamel and dentin.^3^

The calcifying odontogenic cyst (COC) is a rare benign odontogenic lesion that exhibits both cystic and neoplastic features. It has a highly diverse histopathologic characteristic.^4^ In 1962, Gorlin^5^ described the first case of COC as a separate entity thus the frequent use of the term Gorlin cyst for this lesion. COC has a wide variety of clinical and histopathologic manifestations, and debate about the true nature of this lesion is still ongoing.^4^, ^6^ Following several classifications and reclassifications, the WHO in 2005 recommended and classified it as calcifying cystic odontogenic tumor (CCOT).^6^ In the 2017 classification, the cystic form of the lesion was classified as a developmental cyst whereas the solid form was classified as a mixed odontogenic tumor.^7^

The combination of COC and ameloblastic fibro‐odontoma has been reported before. However, this combination is extremely rare. This paper reports the first case of developing odontoma as a new entity arising from calcifying odontogenic cyst.

## 2 CASE REPORT

**FIGURE 1:**
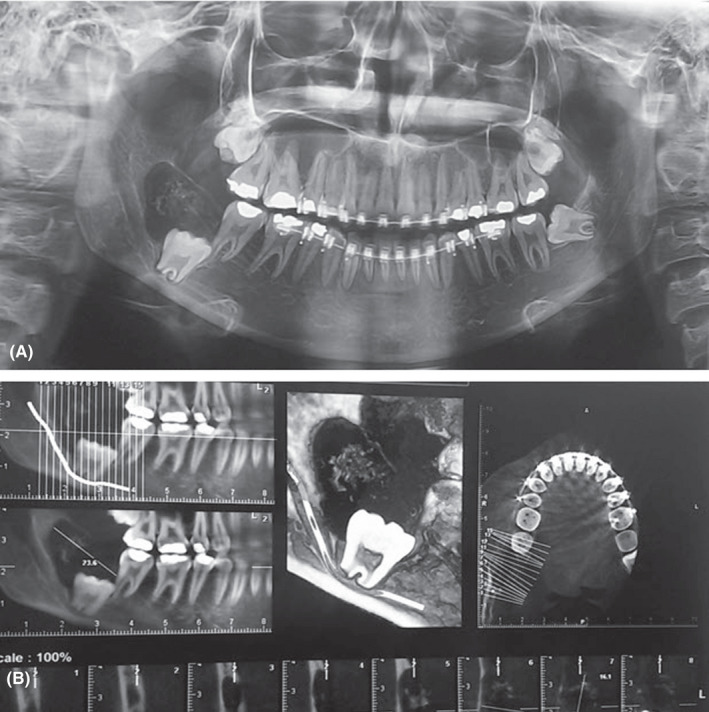
Panoramic radiograph of the lesion that shows well‐defined radiolucent lesion contained areas of radiopacity around the crown of an unerupted third molar (A). Cone beam computed tomography (CBCT) view of the lesion (B)

A 17‐year‐old female patient was referred to the Oral and Maxillofacial Pathology Department, Tehran University of Medical Sciences, Tehran, Iran, for consultation and diagnosis of a lesion on the right side of her mandible. The previous histopathologic diagnosis of the lesion was ameloblastoma. Before the incisional biopsy, the patient's chief complaint was a swelling in the right posterior mandible for 2 weeks. There was no facial asymmetry. In the intraoral examination, a slight swelling was observed in the right retromolar area. The swelling was non‐tender and was firm in consistency. Panoramic and CBCT radiographs showed a well‐defined radiolucent lesion that contained areas of radiopacity around the crown of an unerupted third molar. The margins of the lesion were sclerotic (Figure 1).

**FIGURE 2:**
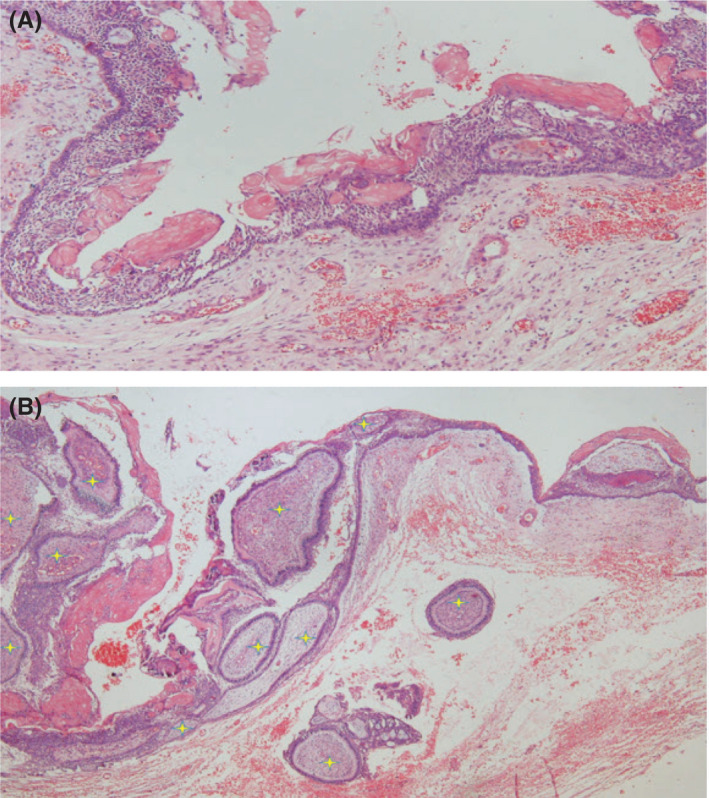
Histopathologic feature of the epithelial lining of COC with prominent ghost cells (×20; A). Sections of tooth buds in the epithelium and also in the cyst wall (×10; B)

**FIGURE 3:**
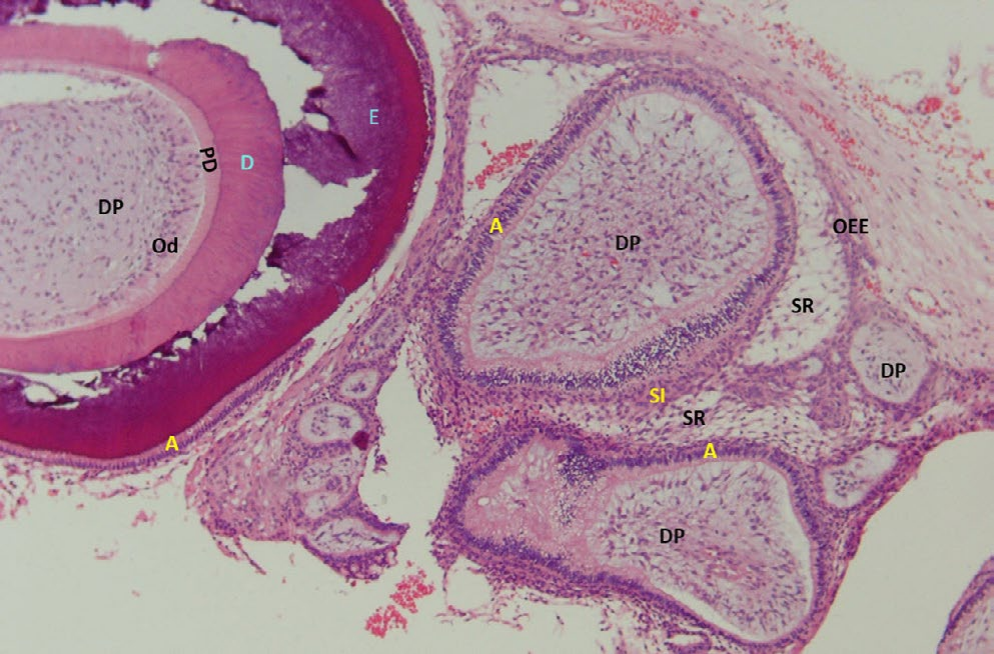
Sections of numerous tooth buds in different stages. Dental papilla (DP); stellate reticulum (SR); stratum intermedium (SI); outer enamel epithelium (OEE); ameloblasts (A); odontoblasts (Od); enamel (E); tubular dentin (D; ×200)

**FIGURE 4:**
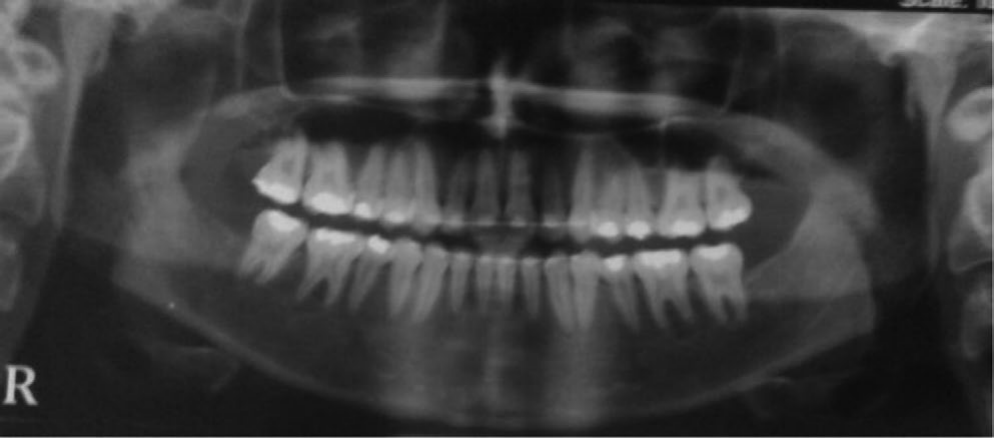
One‐year postoperative radiograph

In the review of the microscopic slides, a cystic lesion lined by an odontogenic epithelium which consists of cuboidal to columnar basal cells with hyperchromatic nuclei demonstrating reverse polarity was observed. The superficial epithelial cells were loosely arranged and resembled the stellate reticulum of enamel organ. Aggregations of ghost cells were notable within the epithelium (Figure 2A). Sections of numerous tooth‐germ structures consisting of nests and islands of enamel organ and dental papilla were also evident in the cyst wall. In some areas, tooth buds appeared to originate from the cystic epithelium (Figure 2B). There were also sections of developed tooth structures consisting of mature enamel cap and tubular dentine (Figure 3). An area of ectomesenchyme‐like cell proliferation similar to dental papilla was also seen. Based on these histopathologic findings, the diagnosis was developing odontoma arising from a calcifying odontogenic cyst. The lesion was treated by conservative excision and there was no recurrence after 1‐year follow‐up (Figure 4).

## 3 DISCUSSION

Unlike solitary tumors, hybrid tumors are rare. However, 25% of cases of calcifying odontogenic cyst (COC) occur concurrently with other odontogenic lesions and the most common concurrent lesion seen in this association is odontoma.^6^ There are very few cases of ameloblastic fibro‐odontoma (AFO) associated with COC, and the first reported case was published in 1987.^8^

Developing odontoma was formerly called AFO until 2017 when WHO changed its name.^1^ This report is the first case of developing odontoma as a new entity arising from calcifying odontogenic cyst. Although the last WHO classification of odontogenic tumors considered the presence of dental hard tissue structures within an ameloblastic fibroma‐like tissue as early stage of a developing odontoma, there are still some argument regarding whether all AFO represent early stage of odontoma. Soluk Tekkesin and Verde suggested that a combination of a cut‐off age over 13.5 and lesion size below 2.1 cm can suggest a developing odontoma. Also, the presence of ameloblastic fibroma‐like tissue in the periphery of the lesion, lobular arrangement of stromal component in a way that hypercellular areas being located mostly around the epithelial component, a well‐developed ameloblastic epithelium and also the presence of ghost cells and cystic structures can serve as histological clues that helps to differentiate developing odontoma from AFO.^9^ Except for histopathologic findings, this concept also revealed that our case represents a developing odontoma.

Developing odontoma is a rare odontogenic tumor. It shows proliferation of odontogenic epithelium and primitive ectomesenchymal tissue in association with tooth structure.^2^ It accounts for about 3% of odontogenic tumors and usually occurs in patients younger than 20 years old.^3^ Over 75% of developing odontomas are located in the mandible and 67% of these are located in the posterior mandible. Over 95% of developing odontomas are associated with impacted permanent teeth and present clinically as painless slow‐growing mass. Radiographically, developing odontoma depicts a clearly defined mixed unilocular or multilocular lesion with various amounts of radiopaque calcifications.^10^

Calcifying odontogenic cyst is a rare odontogenic lesion presented as a painless slow‐growing lesion with predilection for the anterior region of the jaws. It affects both maxilla and mandible equally.^11^ It also occurs equally in males and females and shows no race predilection. Intraosseous COC lesions are more common than the peripheral forms.^2^ COC affects patients between 5 to 92 years and the age of peak incidence is between the second and sixth decade of life.^12^ Radiographically, it shows either well‐defined unilocular or multilocular radiolucencies and sometimes diffuse radiopacities.^11^

Calcifying odontogenic cyst may occur in association with an impacted tooth. The distinguishing histopathologic feature of COC is the presence of ghost cells, which may calcify, in an ameloblast‐like epithelium.^11^ The mechanism of ghost cell formation is not fully understood yet. Ghost cells were previously considered a form of normal or aberrant keratinization of odontogenic epithelium, or as a simple cellular degeneration, or as enamel matrix. Some believe that ghost cells may be due to ischemic degeneration.^13^ It can arise in association with other odontogenic lesions such as odontoma, ameloblastic fibroma, and ameloblastic fibro‐odontoma (AFO). The most frequent concurrent odontogenic lesion with COC is odontoma.^6^ Association of COC with ameloblastic fibro‐odontoma is extremely rare and so far, only three cases have been reported.^8^, ^14^, ^15^

The first case of calcifying odontogenic cyst with ameloblastic fibro‐odontoma was reported by Farman, et al in 1978.^8^

Matsuzaka, et al reported a case of ameloblastic fibro‐odontoma arising from a calcifying odontogenic cyst in 2001. The patient was a 23‐year‐old male with the chief complaint of painful swelling on the left mandibular molar region. It was a multilocular mixed lesion in the panoramic radiograph. Tooth impaction was also evident.^14^

Lee et al., in 2014 reported calcifying odontogenic cyst associated with ameloblastic fibro‐odontoma of the anterior mandible in a 4‐year‐old girl. The chief complaint of the patient was swelling swollen jaw. It had caused tooth displacement. It was a unilocular mixed lesion that caused root resorption and cortical perforation. The lesion was also around an impacted tooth.^15^

Apart from these two combinations of COC with ameloblastic fibro‐odontoma, there were also two other case reports of COC with two other lesions forming a combination of three odontogenic lesions.^16^, ^17^ Imani, et al in 2017 reported a hybrid odontogenic tumor in a 14‐year‐old boy with a painless lesion in the left maxillary canine which was without expansion. The lesion had a mixed radiolucent‐radiopaque appearance with a well‐defined border in the panoramic radiograph. In histopathologic examination, combination of three odontogenic lesions including calcifying odontogenic cyst, complex odontoma, and ameloblastic fibro‐odontoma were evident.^16^

On the contrary, Phillips also reported a hybrid odontogenic tumor in a 7‐year‐old boy which consisted of ameloblastic fibro‐odontoma, COC, and adenomatoid odontogenic tumor. The lesion was discovered on panoramic examination by his dentist as a radiolucent lesion around unerupted succedaneous teeth extending from the deciduous left second molar to the left lateral incisor.^17^

The mechanism that causes the occurrence of two odontogenic lesions together is not well known. Nevertheless, various theories have been proposed to explain the phenomenon including a transformation of one lesion into another, a collision of two separate lesions, and an inductive effect of one lesion on another.^18^ According to the histopathologic findings, the first and third theories seem to be more plausible in our case.

## 4 CONCLUSION

We report an extremely rare case of developing odontoma arising from calcifying odontogenic cyst. This report highlights the importance of accurate diagnosis that influences the treatment plan.

## CONFLICT OF INTEREST

The authors declare no conflict of interest.

## AUTHOR CONTRIBUTIONS

AA gathered the data from the literature and wrote the first draft of the manuscript. MMKh supervised during the whole process and prepared the images for the manuscript. MMKh and NM performed pathologic diagnosis. NM approved the final manuscript.

## ETHICAL APPROVAL

Written informed consent was obtained from the patient and her parents.

## CONSENT

Published with written consent of the patient.

## Data Availability

The data that support the findings of this study are available on request from the corresponding author. The data are not publicly available due to privacy or ethical restrictions.
